# Comparison of the Properties of Acellular Matrix from the Skins of Cod (*Gadus morhua*) and Tilapia (*Oreochromis mossambicus*)

**DOI:** 10.3390/jfb16030081

**Published:** 2025-02-25

**Authors:** Yu Liu, Zeyu Wei, Rui Duan, Ke Wang, Tianyue Xu, Binxian Mao, Junjie Zhang

**Affiliations:** 1 Jiangsu Key Laboratory of Marine Bioresources and Environment, Jiangsu Ocean University, 59 Cangwu Road, Lianyungang 222005, China; 2022220843@jou.edu.cn (Y.L.); 2020221232@jou.edu.cn (Z.W.); 1997000036@jou.edu.cn (R.D.); 2022220807@jou.edu.cn (B.M.); 2 College of Marine Food and Bioengineering, Jiangsu Ocean University, 59 Cangwu Road, Lianyungang 222005, China; 2021221254@jou.edu.cn (K.W.); 2021221212@jou.edu.cn (T.X.); 3 Co-Innovation Center of Jiangsu Marine Bio-Industry Technology, Jiangsu Ocean University, 59 Cangwu Road, Lianyungang 222005, China; 4 Jiangsu Institute of Marine Resources Development, Jiangsu Ocean University, 59 Cangwu Road, Lianyungang 222005, China

**Keywords:** cod skin, tilapia skin, acellular matrix, wound healing, biomaterials

## Abstract

Acellular tissue matrices of fish skin origin are highly promising materials for tissue engineering due to their low biological risks and few religious restrictions. The main component of acellular fish skin matrices (AFSMs) is collagen, but collagen properties significantly differ between marine and freshwater fish. Although the characteristics of acellular matrices may vary, relevant reports about them are few. In this study, we used cod and tilapia fish skin as raw materials to prepare acellular matrices with low DNA content (≤50 ng/mg) and low endotoxin. They were denoted as C-AFSM (cod) and T-AFSM (tilapia) and had endotoxin removal rates of 92.47% and 96.73%, respectively. Their physicochemical properties, cytotoxicity, and wound healing effects were evaluated and compared. Scanning electron microscopy images showed that C-AFSM and T-AFSM had collagenous meshwork and high porosity. They also did not induce skin irritations. Their proliferation rates on mouse fibroblasts at 36 h were 192.21% ± 33.25% and 162.89% ± 36.47%, respectively. The wound healing effect of C-AFSM was faster than that of T-AFSM group (7 and 14 days: 45.3% ± 5.99% and 93.77% ± 1.58% for C-AFSM and 39.7% ± 2.84% and 93.35% ± 1.1% for T-AFSM, respectively). Therefore, the two acellular fish skin matrices can be used as tissue-engineering materials for wound repair, with C-AFSM being more effective than T-AFSM.

## 1. Introduction

Acellular tissue matrix (ACTM) is biodegradable, provides a suitable space for cell growth [1], and has low immunogenicity. It can promote tissue repair by preserving bioactive molecules [2]. Thus, ACTM as a natural biomaterial is an ideal scaffold for skin wound repair [3,4]. Currently, ACTM is derived mostly from human, bovine, or porcine tissue material [5,6], but interest in acellular tissues of non-mammalian origin is increasing due to religious restrictions and the risk of transmitting zoonotic diseases (e.g., cases of bovine spongiform encephalopathy, swine influenza, and foot-and-mouth disease) [7]. Among non-mammalian tissues, the extracellular matrix (ECM) of fish skin is an excellent choice for human skin replacement due to its high collagen content, low antigenicity, and excellent three-dimensional network structure, as well as its specific bioactive components [8,9,10]. Although some acellular fish skin matrices (AFSMs) are used for clinical treatment [11,12], research on acellular matrices of fish origin remains in the preliminary stages [2,13].

*Gadus morhua* (Cod), a cold-water fish extensively distributed throughout the world’s oceans, is one of the most commercially important food species globally [14]. Cod skin is high in collagen and has a balanced protein–lipid composition. In lipids, ω-3 polyunsaturated fatty acids are known to promote wound healing [15]. Cod skin-based ACTM-Kerecis™ (Kerecis, Isafjordur, Iceland) is the first commercialized fish ECM product approved by the US FDA [16] and is an excellent alternative to tissue scaffolds.

Tilapia (*Oreochromis mossambicus*), a tropical freshwater fish native to Africa, is highly recommended by the United Nations Food and Agriculture Organization as an excellent fish species for aquaculture. Tilapia is characterized by high adaptability and high disease resistance [17]. Tilapia skin is available from wide-ranging sources and produced in large quantities, so it is a rational raw material for AFSM preparation [11].

The main component of AFSM is collagen. Significant differences in the nature of collagen, especially denaturation temperature [18,19], exist between marine and freshwater fish sources (tilapia skin, 35.2 °C; cod skin, 16.8 °C) [20,21]. These features may lead to different properties of their acellular matrix. In a previous study [9], we found that silver carp (freshwater fish) acellular matrix (SC-AFSM) has excellent biological properties. Due to the lower denaturation temperature of cod collagen, we hypothesized that cod skin acellular matrix (C-AFSM) may have better performance in terms of biocompatibility. Therefore, we decided to compare tilapia skin acellular matrix (T-AFSM) with C-AFSM. In the field of biology, cod and tilapia as the raw materials are more researched, and the referable data and comparisons are readily available. However, reports on the differences in AFSMs between marine and freshwater fish are few [22].

In the present study, cod skin (CK) and tilapia skin (TK) were used as raw materials to prepare low-DNA-content and low-endotoxin cod skin acellular matrix (C-AFSM) and tilapia skin acellular matrix (T-AFSM), respectively. The structural and mechanical properties of the two types of AFSMs were compared, and cytotoxicity and trauma healing effects were evaluated and compared. We aimed to analyze the differences in AFSM properties between marine and freshwater fishes and to provide basic information for expanding the source of novel tissue-engineering materials.

## 2. Materials and Methods

### 2.1. Materials

Trypsin was purchased from Beijing Solebo Technology Co., Ltd. (Beijing, China). Sodium dodecyl sulfate (SDS) and TritonX-100 were purchased from Sigma Aldrich Trading Co., Ltd. (Shanghai, China). A DP705 Magnetic Bead Method Universal Genomic DNA Extraction Kit was purchased from Tiangen Biochemistry and Technology Co., Ltd. (Beijing, China). An L00350 ToxinSensor^TM^ Endotoxin Detection Kit was purchased from Nanjing Kingsley Biotechnology Co., Ltd. (Nanjing, China). A CCK-8 kit was purchased from Sangong Biological Engineering Co., Ltd. (Shanghai, China). All media and reagents were purchased from Sangon Biological Engineering Co., Ltd. (Shanghai, China). All reagents were analytical-grade. Cod skin was purchased from Qingdao Jinheshun Aquatic Products Co., Ltd. (Qingdao, China) and tilapia skin was purchased from Guangzhou Longzhirun Aquatic Products Processing Co., Ltd. (Guangzhou, China). The two fish skins were removed from the non-skin components, rinsed three times in tap water, and stored at −20 °C for later use. Decellularized matrix (commercial product, CP) was purchased from Jiangsu Unitrump Biomedical Technology Co., Ltd. (Nantong, China).

### 2.2. Preparation of AFSM

CK and TK were processed with reference to the protocol for the preparation of chub AFSM by Zhang et al. [9]. The skins were thawed, rinsed in distilled water, and infiltrated with 500 ppm penicillin–streptomycin in sterile phosphate-buffered solution (S-PBS) at 8 °C for 0.5 h. After that, the skins were treated by immersion in TritonX-100 and 0.5% SDS for 10 h and 1 h, respectively, and then trypsinized for 12 h and finally 1% TritonX-100 for 12 h to remove endotoxins.

The AFSMs were prepared in sterile bags. They were freeze-dried in a sterile room (TF-LFD-6A-Freeze dryer, Shanghai Tuodan Machinery Equipment Co., Ltd., China) and irradiated and sterilized (Clean Bench-Suzhou CNNC Huadong Irradiation Co., Ltd., China).

### 2.3. Acellular Process

#### 2.3.1. Determination of DNA Content

Fish skin (40 mg) was taken and cut into small pieces; 300 μL of tissue digest and 20 μL of Proteinase K was added, and the samples were ground with an electric homogenizer (OSE-Y30, Tiangen Biochemical Technology Co., Ltd., Beijing, China) for about 20 s. The samples were heated in a water bath at 65 °C for 1 h. The samples were cut up and weighed. DNA was extracted from the samples using a DNA extraction kit (Q5000, Quawell Inc., San Jose, CA, USA), and DNA was determined. The DNA content was calculated according to the formula

$$Y\left(\frac{ng}{mg}\right)=100\times \frac{N}{m}$$

in which 100 is the volume of dilution (μL), N is the measured DNA content (ng/μL), m is the dry weight of the sample (mg), and Y is the total DNA content in the acellular matrix.

#### 2.3.2. Histological Staining Analysis

Samples of 1 cm × 0.5 cm were taken, fixed in 4% paraformaldehyde for 24 h, dehydrated in 50%, 70%, 80%, 90%, 95%, and 100% ethanol sequentially for 1 h, made transparent in xylene for 1 h (0.5 h × 2), dipped in wax three times for 1 h each time, and embedded in paraffin wax with a melting point of 52–60 °C. Sections were cut with a rotary slicer (HistoCore BIOCUT, Leica Biosystems, Wetzlar, Germany) at a thickness of 5 μm, and the cutting temperature was set at 43 °C. Sections were stained with hematoxylin and eosin and examined by light microscopy. Blue or purple areas indicate the presence of DNA, while pink areas represent protein.

### 2.4. Determination of Endotoxin Content

AFSMs of 6 cm^2^ were taken, weighed, and placed in a sterile centrifuge tube, and sterile, distilled water was added at a sample surface area/extract ratio of 6:1 (*s*/*v*) for immersion at 25 °C for 2 h. The endotoxin was determined using an endotoxin detection kit. The actual endotoxin content was calculated according to the formula

E(EU/g)=V×(0.7006×A+0.0523)/M

in which V is the volume of sterile distilled water added (mL), and M is the dry weight (g) of the 6 cm^2^ sample.

### 2.5. Characterization of the Physical Properties of AFSM

#### 2.5.1. Microstructure Analysis

C-AFSM and T-AFSM were cut into 4 mm × 1 mm rectangles for the observation of the sides, and after vacuum spraying, the accelerating voltage was set at 15.0 kV. The morphology of the acellular tissue matrix was observed under a scanning electron microscope (SIGMA300, ZEISS, Baden-Württemberg, Germany) by selecting the appropriate magnification [23]. The thickness of the 20 samples was labeled on average, and the thickness was calculated by taking the average value.

#### 2.5.2. Determination of Porosity

The method of Yang et al. [24] was referred to with appropriate modifications. A 1 mm × 1 cm sample was weighed, placed in 50 mL of 75% ethanol for 1 h, and degassed with a vacuum pump until no bubbles escaped. The beaker containing ethanol and acellular tissue matrix was weighed, the acellular tissue matrix was removed, and the remaining ethanol and the mass of the beaker were weighed. The porosity was calculated according to

$$P(\%)={(m}_{2}-m_{3}-m_{1})/(m_{2}-m_{3})\times 100\%$$

where m_1_ was the initial mass of the sample, m_2_ was the mass of the beaker containing the ethanol and acellular matrix, and m_3_ was the mass of the remaining ethanol and the beaker.

#### 2.5.3. Determination of Tensile Stress

The tensile stress of the samples was determined using the method in GB/T 1040.3-2006 [24]. The samples were made into a dumbbell shape of 50 mm × 5 mm, rehydrated with distilled water, and then measured with a mass tester (TMS-PRO type, FTC Corporation, Alexandria, VA, USA), with stretching at a speed of 10 mm/min until the samples broke, and the measurement was repeated three times to take the average value. The stress values were calculated according to

σ(Mpa)=F/A

in which F is the corresponding load measured, and A (1.55 mm^2^) is the original cross-sectional area of the specimen.

### 2.6. Biocompatibility Evaluation

#### 2.6.1. Skin Irritation Test

Healthy male Kunming mice (KM mice, SPF grade, 4–5 weeks, 20–25 g) were purchased from Specific (Beijing) Biotechnology Co., Ltd. and the experiments were started after 7 days of acclimatization feeding. The backs of rats were shaved, four 1 cm × 1 cm areas were divided, the samples were cut to a uniform size and pasted onto the areas, and the skin surface was observed for redness, swelling, rashes, and other undesirable phenomena.

#### 2.6.2. Cytotoxicity Assay

AFSM cytotoxicity was evaluated by referring to the method of Cai et al. [25] with appropriate modifications. Mouse fibroblasts (3T3 cells) were used to culture in Dulbecco’s Modified Eagle’s Medium (DMEM) (containing 10% fetal bovine serum, 1% penicillin/streptomycin) in a 37 °C CO_2_ incubator (Galaxy S, RS Biotech, Borehamwood Hertfordshire, UK) and were passaged every 2–3 days.

The C-AFSM and T-AFSM of 6 cm^2^ were put into a test tube, and 1 mL of DMEM was added at the sample surface area/extraction solution ratio of 6:1 (*s*/*v*), and the extract was incubated in a 37 °C CO_2_ incubator for 24 h to make the extraction solution. The extract was diluted with DMEM to 50%, 25%, and 12.5% concentration, respectively, and then prepared for use.

3T3 cells were inoculated in 96-well plates at a density of 5000/well and incubated at 37 °C for 24 h. The plates were divided into three groups: blank group (3T3 cells), negative control group (DMEM), and experimental group (each concentration of the extract). Each group was spiked with 10 μL and incubated for 24 h. OD values were determined at 450 nm using a CCK-8 kit to obtain formazan, which produces an orange-yellow color by reduction reaction (Synergy HT type enzyme labeling instrument, Bio-Tek, USA). The relative cell proliferation rate was calculated according to the formula

$$Relative\;cell\;proliferation\;rate(\%)=({OD}_{b}-{OD}_{c})/({OD}_{a}-{OD}_{c})\times 100\%$$

in which OD_a_ is the absorbance value of the negative control group (medium + cells), OD_b_ is the absorbance value of the experimental group, and OD_c_ is the absorbance value of the blank control group (cells).

In 96-well plates, 3T3 cells were inoculated at a density of 2000/well and cultured at 37 °C for 24 h. The culture medium was discarded, and 100 μL of two substrate extracts and DMEM medium was added to culture for 24 h, 48 h, and 5 d, respectively. Then, the cells were stained with AO/EB dye, and the cell proliferation status was observed under an inverted fluorescence microscope.

#### 2.6.3. Wound Healing Test

Healthy male Kunming mice (KM mice, SPF-grade, 4–5 weeks, 20–25 g) were purchased from Specific (Beijing) Biotechnology Co., Ltd., and the experiments were started after 7 days of acclimatization feeding. The KM mice were randomly divided into three groups: blank group (no treatment, BK), positive control group (sodium carboxymethyl cellulose dressing purchased from Jiangsu Unifrump Biomedical Technology Co., Ltd. (Nantong, China). CP), and experimental group (C-AFSM, T-AFSM). The whole experiment was conducted in accordance with the Regulations on the Administration of Experimental Animals formulated by the National Science and Technology Commission and the Regulations on the Administration of Medical Experimental Animals formulated by the Ministry of Health.

Mice were bound and injected intraperitoneally with 0.3 mL of Tribromoethanol, and after successful anesthesia, body hair on the back of mice was removed with an electric shaver. The backs of the mice were sterilized with iodophor, deiodinated with 75% ethanol, and the skin covering the muscles was removed by forming two total injury ports on the backs of the mice with a skin punch of 8 mm in diameter. The wounds were sterilized with 75% ethanol, and the limbs of the mice were abducted and fixed to the surgical plate with rubber bands. A silica gel ring with an inner diameter of 8 mm was secured to the edges of the wounds with a 4-0 nonabsorbable suture. The C-AFSM, T-AFSM, and CP C-AFSM, T-AFSM, and CP were prepared to the wound size and soaked in saline for 0.5 h, covering the wound surface, and the silicone ring was observed daily for the first week to see if it was detached and replenished.

Postoperative mice were housed in single cages, and the performance of the mice was observed and recorded daily, including whether there was any death, daily feeding, water intake, activity, mental status, and other indicators. The wounds were photographed at 1, 7, 14, 21, and 28 days postoperatively, and the wound healing area was analyzed with Image J. The wound healing rate was calculated using the formula of the healing area of the wound. The wound healing rate was calculated according to the formula

Wound healing rate (WHR, %) = Healed area/original wound area × 100%

A batch of mice were executed on days 1, 7, 14, 21, and 28, and histological analysis of the repair area was performed by HE staining.

### 2.7. Statistical Analysis of Data

Data were analyzed using SPSS 23 (IBM, Amonk, NY, USA) and Origin 2021 (Origin Laboratories Inc., Denver, CO, USA) software. Results were expressed as the mean ± standard deviation (SD). Unless otherwise stated, comparisons between the two groups were analyzed using one-way analysis of variance (ANOVA). The differences were statistically significant, defined as *p* < 0.05, *p* < 0.01.

## 3. Results and Discussion

### 3.1. DNA Residue and Endotoxin Content

**Figure 1:**
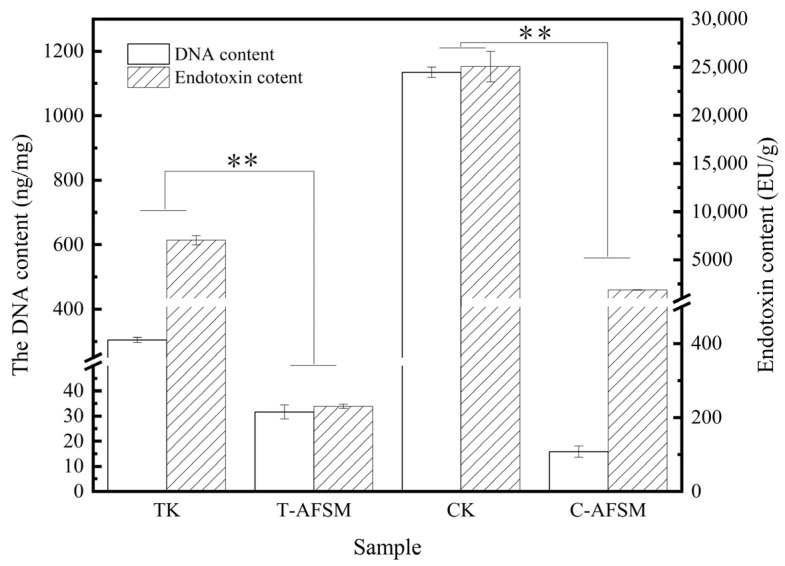
DNA residues and endotoxin content of C-AFSM and T-AFSM. CK: cod skin; C-AFSM: cod acellular fish skin matrix; TK: tilapia skin; T-AFSM: tilapia acellular fish skin matrix. ** *p* < 0.01 indicated extremely significant difference between the two groups.

The results in Figure 1 show that CK and TK can reach below the medical industry-recognized limit of 50 ng/mg of DNA residue after decellularization [26] (C-AFSM, 15.87 ± 2.3 ng/mg; T-AFSM, 31.64 ± 2.78 ng/mg).

The results of T-AFSM are similar to those reported by Kamalvand [27], who found a DNA residue of 30.5 ± 3.8 ng/mg after tilapia skin decellularization using a PBS solution of 1 M NaCl, 50 mM trimethylaminomethane, and 10 mM ethylene diamine tetraacetic acid. Meanwhile, the endotoxin removal rates were 96.73% and 92.47% for C-AFSM and T-AFSM, respectively.

**Figure 2:**
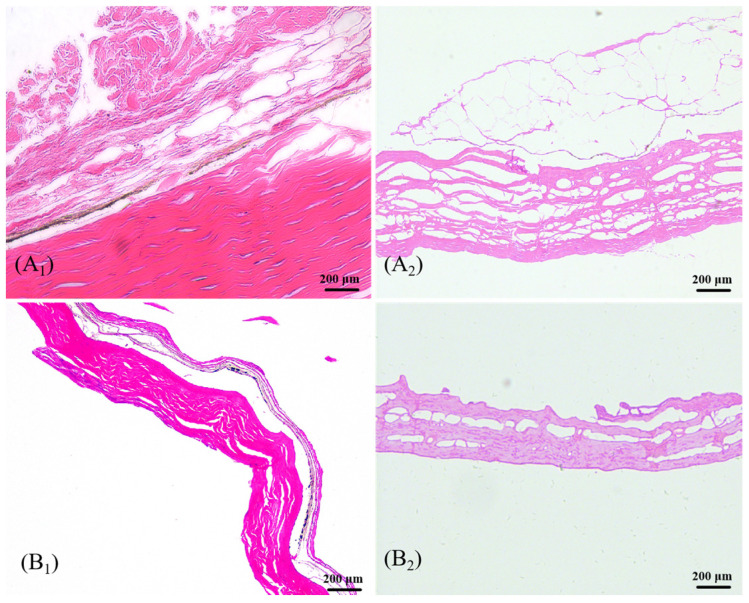
Results of HE staining of T-AFSM and C-AFSM. (**A_1_**): CK; (**A_2_**): C-AFSM; (**B_1_**): TK; (**B_2_**): T-AFSM.

Figure 2 shows the HE staining images. CK and TK had no obvious nuclei after decellularization treatment, and C-AFSM and T-AFSM retained the original regular collagen fiber structure. However, compared with the complete, dense, and porous ECM network of silver carp acellular fish skin matrix (SC-AFSM) [9], some damage was inflicted to the ECM network of T-AFSM, and the delamination of collagen fibers of C-AFSM was clearer [11]. This finding is consistent with the results of the HE staining of tilapia after decellularization treatment using Triton X100 0.25% *w*/*v* in the report of Kamalvand et al. [27] The acellular matrix was prepared with physical methods. Although the cells can be completely removed, the structure of the matrix was usually greatly damaged [28].

### 3.2. Physical and Chemical Characterization

#### 3.2.1. Microstructure Analysis

**Figure 3:**
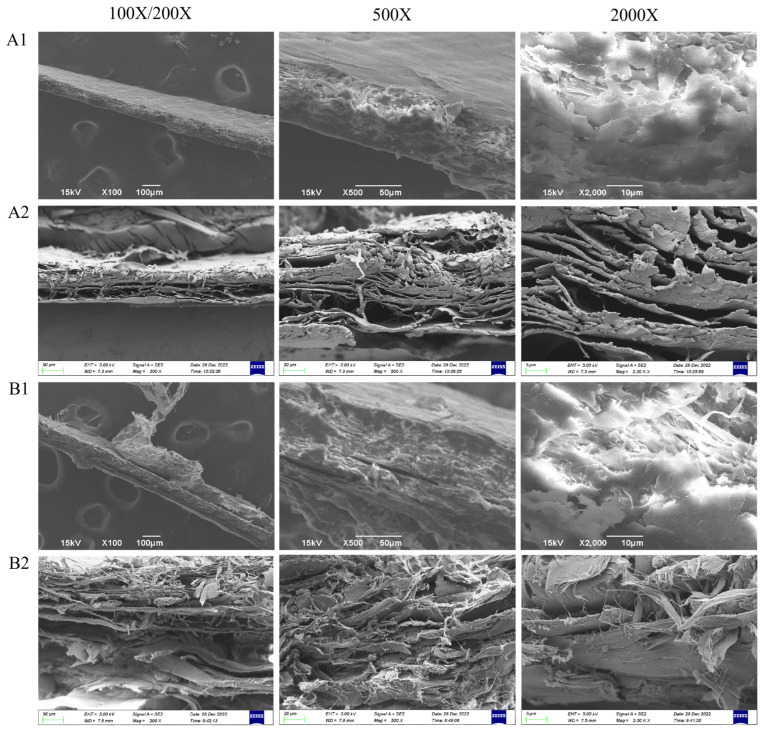
SEM results of the side of C-AFSM and T-AFSM. (**A1**): CK; (**A2**): C-AFSM; (**B1**): TK; (**B2**): T-AFSM.

Figure 3 shows lateral SEM images of CK, C-AFSM, TK, and T-AFSM. After decellularization treatment, the structure of C-AFSM and T-AFSM was looser than that of CK and TK, and the interlayer gap of collagen fibers increased significantly, forming a loose ECM meshwork. After the decellularization of tilapia skins by Liu [29] and other researchers [30,31,32], the outer surface of the acellular matrix becomes relatively smooth, and its inner part has a typical state of longitudinal and transverse arrangement.

#### 3.2.2. Porosity

The porosity of C-AFSM (90.57% ± 0.35%) was higher than that of silver carp (*Hypophthalmichthys molitrix*) acellular fish skin matrix, SC-AFSM (79.64% ± 0.17%) [9], whereas the porosity of T-AFSM (83.7% ± 1.36%) was slightly higher than that of SC-AFSM. Thus, the porosity of the acellular fish skin matrices prepared from the two types of freshwater fish skin (tilapia and chub fish) (T-AFSM and SC-AFSM) had similar porosity but lower than that of C-AFSM. Li et al. [11] found that the hydroxyproline content of fish skin collagen in marine fish is lower than that of freshwater fish, leading to a more thorough decellularization treatment of marine fish. When the pH was between 6 and 8, the relative solubility of collagen in marine fish was higher than that in freshwater fish [33], so collagen leaching from CK was higher than that of TK during the acellular process. Consequently, the porosity in C-AFSM was higher, consistent with the results of scanning electron microscopy.

#### 3.2.3. Tensile Strength and Elongation at Break

Table 1 shows the thickness of CK. It decreased from 0.24 ± 0.02 mm to 0.17 ± 0.03 mm after decellularization treatment, a decrease of about 29.17%. Conversely, no significant difference existed in the thickness of TK. The mechanical properties of both AFSMs were altered. The tensile strength of T-AFSM decreased from 17.08 ± 0.62 MPa to 5.79 ± 0.11 MPa, which was slightly higher than that of the acellular matrices of tilapia skin (4.46 MPa) reported by Liu [29]. The elongation at break decreased from 88.37% ± 8.57% to 64.79% ± 5.41%, which was higher than that of SC-AFSM (TS: ±7.49%, EAB: ±17.08%). The thickness and mechanical properties of CK were lower than those of TK, and C-AFSM disintegrated soon after rehydration, rendering the tensile strength and elongation at break impossible to determine. This phenomenon was related to the lower denaturation temperature of collagen in marine fish compared to that of freshwater fish (e.g., cod skin, 16.8 °C; tilapia skin, 35.2 °C) [20,21]. The denaturation temperature of cod skin collagen was lower than room temperature, indicating that collagen underwent degradation and denaturation after rehydration. Li [11] also demonstrated that the hydroxyproline content in marine fish is lower than that in freshwater fish, and that hydroxyproline and GAG affect the acellular product’s physical properties, such as thermal stability and mechanical strength. The relative decrease in mechanical properties was consistent with the electron microscopy results.

### 3.3. Biocompatibility Evaluation of AFSM

#### 3.3.1. Skin Irritation Test

**Figure 4:**
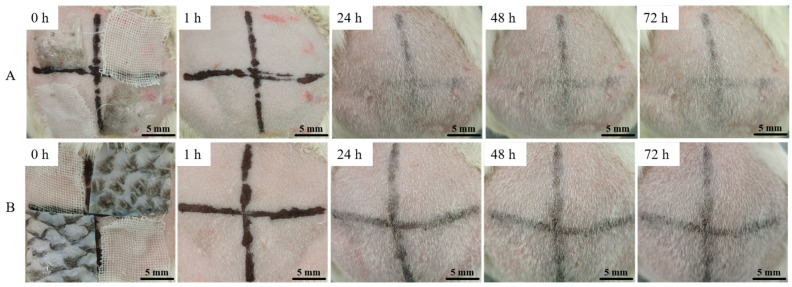
Results of animal skin irritation experiments of T-AFSM and C-AFSM. (**A**): C-AFSM; (**B**): T-AFSM.

The results of skin irritation experiments of C-AFSM and T-AFSM are shown in Figure 4. After removing the dressing sheet and observing the skin contact area at each time point, the control group (gauze) scored 0 points on average, very slight erythema was present in the C-AFSM group (scoring 0.3 points on average), and no erythema existed in the T-AFSM group (scoring 0 points on average). Thus, C-AFSM and T-AFSM did not irritate animal skin.

#### 3.3.2. Cytotoxicity Assay

**Figure 5:**
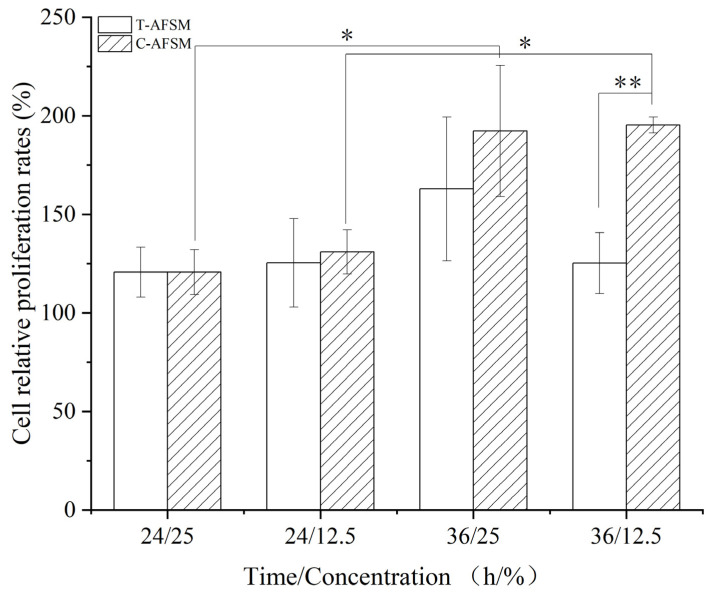
Comparison of relative cell proliferation rates in different AFSMs. C-AFSM: cod acellular fish skin matrix; T-AFSM: tilapia acellular fish skin matrix. * *p* < 0.05 indicated that there was a significant difference between the two groups; ** *p* < 0.01 indicated that the difference between the two groups was extremely significant.

As shown in Figure 5, C-AFSM and T-AFSM were not cytotoxic. With 25% extract concentration, at 24 h, the cellular value-added rate of C-AFSM was 120.7% ± 12.71%, which was almost the same as that of T-AFSM; at 36 h, the cellular value-added rates of C-AFSM and T-AFSM were 192.21% ± 33.25% and 162.89% ± 36.47%, respectively. These values were 71.51% and 42.19% higher than those at 24 h, so C-AFSM had a greater effect on cell proliferation than T-AFSM. With 12.5% extract concentration, from 24 h to 36 h, the results of C-AFSM were close to that with 25% concentration, and the effect on cell proliferation was significant. However, almost no increase occurred for T-AFSM. This result was similar to those of Lau et al. [7] Overall, the cell proliferation rate of C-AFSM was higher than that of T-AFSM, which was attributed to the fact that C-AFSM infiltrated more collagen components than T-AFSM [33]. Moreover, the arginine/glutamine, valine, isoleucine, leucine, and most importantly hydroxyproline in collagen had a pro-proliferative effect on cell proliferation [34].

**Figure 6:**
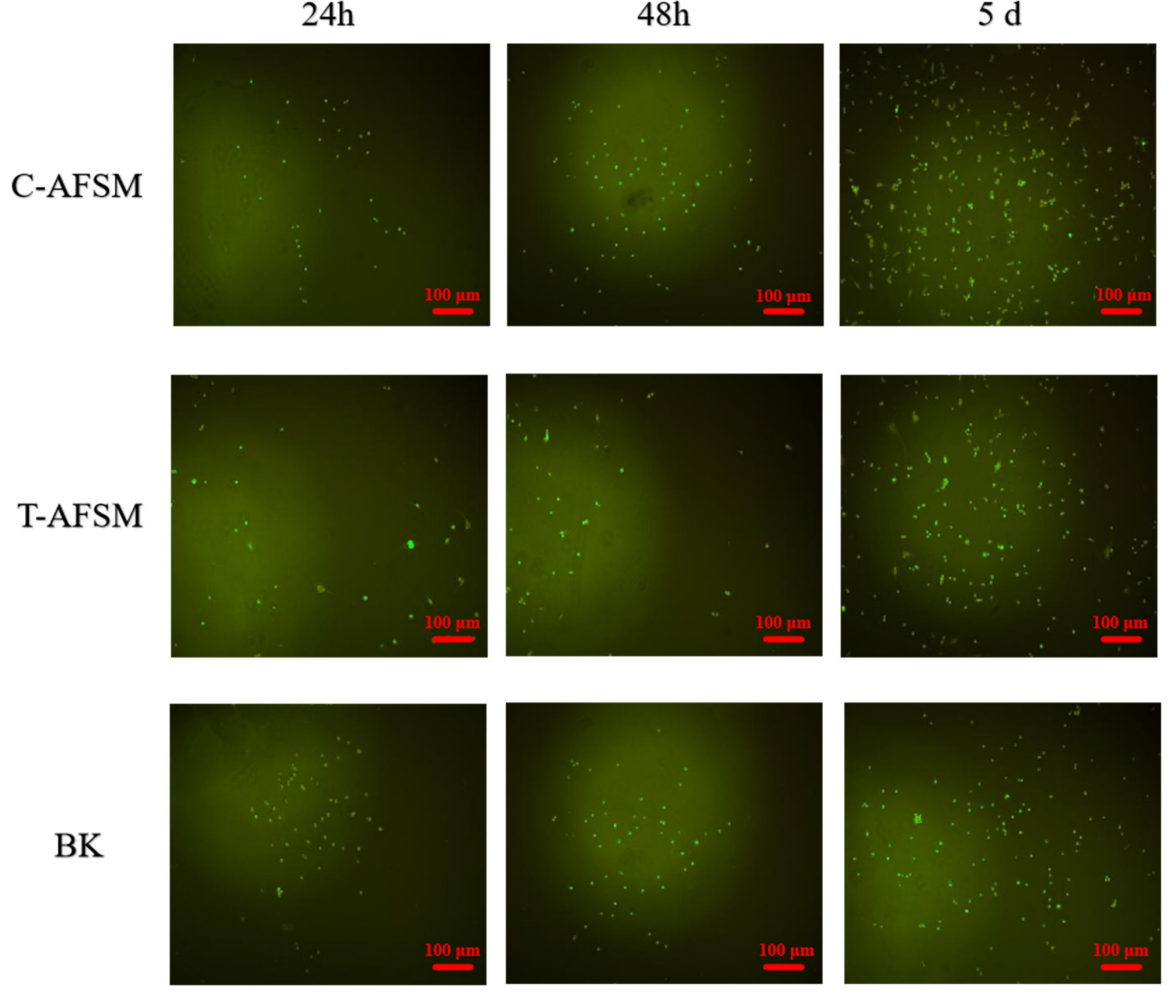
NH3T3 cell proliferation of C-AFSM and T-AFSM after 24 h, 48 h, and 5 d culture. C-AFSM: cod acellular fish skin matrix; T-AFSM: tilapia acellular fish skin matrix; BK: blank control.

Figure 6 shows the observation results of mouse fibroblasts cultured in C-AFSM and T-AFSM extracts for different times. Under the same conditions, C-AFSM extract was better than T-AFSM extract for cell proliferation, which is the same as the cytotoxicity result. No orange fluorescence was observed in both acellular matrices after AO/EB staining, indicating no cell death during the culture process. We further verified that the two acellular matrices were effective in endotoxin removal and that C-AFSM and T-AFSM were not cytotoxic.

#### 3.3.3. Wound Healing Test

**Figure 7:**
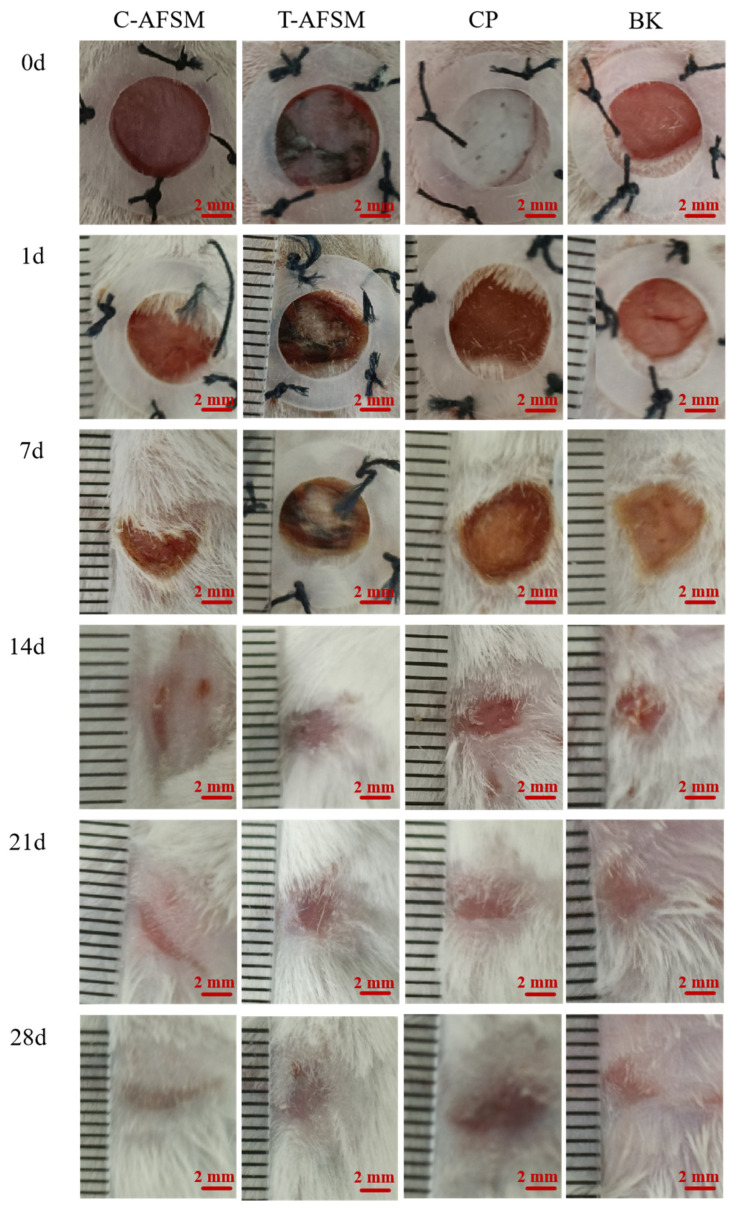
Results of wound healing experiments of C-AFSM and T-AFSM. C-AFSM: cod acellular fish skin matrix; T-AFSM: tilapia acellular fish skin matrix; CP: commercially available product; BK: blank control.

**Figure 8:**
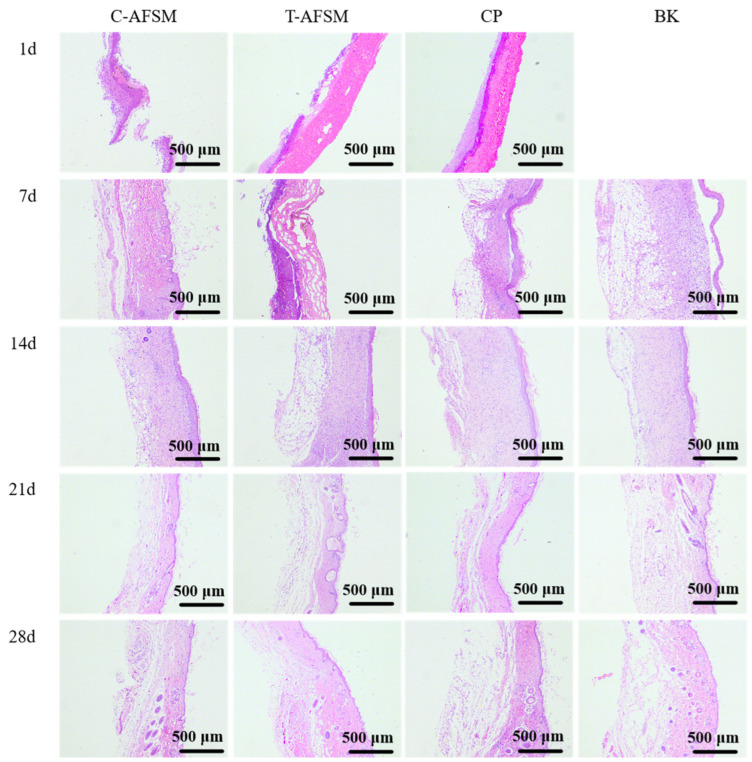
Results of HE staining of wound tissue of C-AFSM and T-AFSM. C-AFSM: cod acellular fish skin matrix; T-AFSM: tilapia acellular fish skin matrix; CP: commercially available product; BK: blank control.

**Figure 9:**
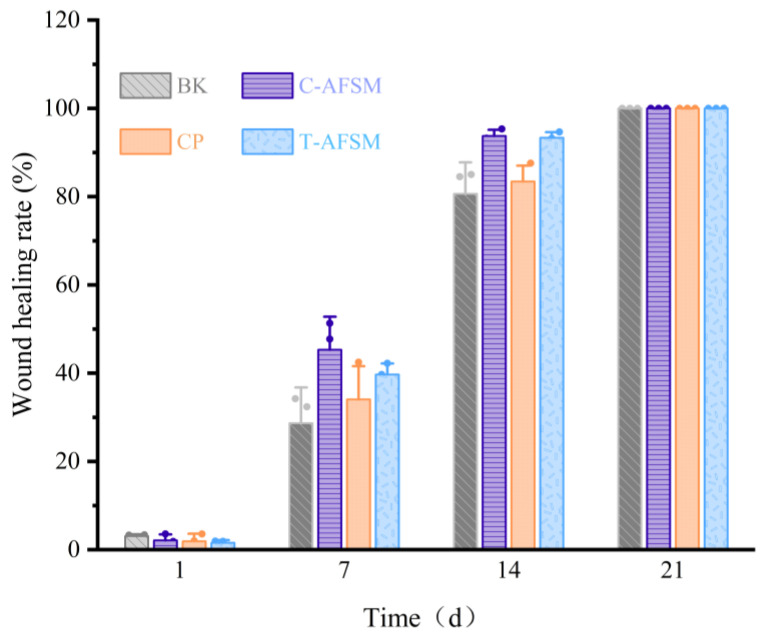
Results of wound healing rate of C-AFSM and T-AFSM. Note: C-AFSM: cod acellular fish skin matrix; T-AFSM: tilapia acellular fish skin matrix; CP: commercially available product; BK: blank control.

During wound healing, all groups of KM mice showed no abnormalities in food intake, water intake, activity, or mental status. Furthermore, no adverse reactions such as redness, swelling, and allergy occurred. As shown in Figure 7, Figure 8 and Figure 9, degradation occurred in the C-AFSM, T-AFSM, and commercially available product (CP) groups on day 1 of implantation. HE staining results on day 1 also showed the fastest degradation in the C-AFSM group. No significant wound healing occurred on day 1 (C-AFSM, 2.12% ± 1.21%; T-AFSM, 1.57% ± 0.69%; CP, 1.91 ± 1.67%; blank control group (BK), 3.08% ± 0.28%). This result was similar to that reported by Li [19], who used Parma mini pig as a trauma model and tilapia skin acellular matrix as dressing material. They found a faster degradation rate of tilapia acellular matrix compared to the negative control group.

On day 7, morphological observations and HE staining results showed that the C-AFSM, T-AFSM, and CP groups had surface crusting and some wound contraction. The undegraded tissue matrix still existed in the T-AFSM group. The healing rates of the C-AFSM, T-AFSM, and CP groups were 45.3% ± 5.99%, 39.7% ± 2.84%, and 34.05% ± 8.43%, respectively. The C-AFSM group showed the best healing effect. A large number of irregular collagen fibers were visible in its HE staining results. C-AFSM had degraded the most on day 1, whereas T-AFSM was partially degraded. C-AFSM showed a faster degradation rate, which may be due to its higher porosity. The surface area effect caused by a different pore morphology of the tissue material and the thickness of the material affected the rate of degradation [35,36]. The results are in accordance with those of previous reports. In addition, marine fish collagen had a lower denaturation temperature, which led to the denaturation of collagen being faster than that of collagen from freshwater fish. The degraded collagen contained growth factor signaling, leading to the expression of TGF-β_1_, VEGF, etc., which could help form epidermal and vascular tissues in the wound area. The wound healing rates were consistent with those on cell proliferation obtained for C-AFSM and T-AFSM.

On day 14, the healing rate of C-AFSM was 93.77% ± 1.58%, followed by the T-AFSM (93.35% ± 1.1%) and CP groups (81.69% ± 3.5%). The effects were better than those of BK (80.66% ± 8.19%). The healing area of the T-AFSM group was similar to that of C-AFSM, which may be due to the nature of the slower decomposition of freshwater fish collagen. It could be inferred that when the wound area is larger and the wound is deeper, T-AFSM might show a better healing effect. Fourteen days after trauma is a critical stage for granulation regeneration. Mild inflammation and mature granulation tissue are generally believed to facilitate wound re-epithelialization and achieve initial wound healing [11].

Wound healing was complete in all groups by 21 days. Hard scab detachment, complete wound healing, tissue formation, and scar tissue contraction were observed on the 21st and 28th days. HE staining results showed the proliferation of granulation tissue, a small number of sebaceous glands and hair follicles, and irregular dense collagen fibers arranged regularly under tension, as well as normal skin tissue structure. Relative scar areas of the C-AFSM and T-AFSM groups were 7.07% ± 2.13% and 8.94% ± 0.91%, respectively, indicating that the scar area after C-AFSM treatment was less than that after T-AFSM treatment.

## 4. Conclusions

Acellular matrices of cod and tilapia skin with low DNA residue and endotoxin content were prepared. Physical and biocompatibility characterizations of C-AFSM and T-AFSM were performed, evaluated, and compared. C-AFSM and T-AFSM had a highly porous ECM collagen meshwork. C-AFSM and T-AFSM were non-skin irritating and non-cytotoxic. C-AFSM was more effective than T-AFSM for cell proliferation and had a faster healing effect. The relative scar area in the C-AFSM group was 20% smaller than that in the T-AFSM group. Therefore, the two acellular fish skin matrices have great potential to be used as tissue-engineering materials for wound repair.

## Figures and Tables

**Table 1:** Measurement results of mechanical properties of C-AFSM and T-AFSM.

Sample	CK	C-AFSM	TK	T-AFSM
Thicknesses (mm)	0.24 ± 0.02	0.17 ± 0.03	0.48 ± 0.02	0.49 ± 0.05
TS (MPa)	13.14 ± 1.39	-	17.08 ± 0.62	5.79 ± 0.11
EAB (%)	39.23 ± 9.67	-	88.37 ± 8.57	64.79 ± 5.41

CK: cod skin; C-AFSM: cod acellular fish skin matrix; TK: tilapia skin; T-AFSM: tilapia acellular fish skin matrix; TS: tensile strength; EAB: elongation at break; -: no value.

## Data Availability

The data that support the findings of this study are available from the corresponding author upon reasonable request.

## Abbreviations

C-AFSM	Cod acellular fish skin matrix
T-AFSM	Tilapia acellular fish skin matrix
SC-AFSM	Silver carp acellular fish skin matrix
CP	Commercially available product
BK	Blank control
ACTM	Acellular tissue matrix
ECM	Extracellular matrix
CK	Cod skin
TK	Tilapia skin
DMEM	Dulbecco’s modification of Eagle’s medium
WHR	Wound healing rate
3T3	Mouse fibroblasts
TS	Tensile strength
EAB	Elongation at break
